# Vulnerability to HIV infection among female drug users in Kathmandu Valley, Nepal: a cross-sectional study

**DOI:** 10.1186/1471-2458-13-1238

**Published:** 2013-12-28

**Authors:** Bhagabati Ghimire, S Pilar Suguimoto, Saman Zamani, Masako Ono-Kihara, Masahiro Kihara

**Affiliations:** 1 Department of Global Health and Socio-epidemiology, Kyoto University School of Public Health, Yoshida Konoe-cho, Sakyo-ku, Kyoto 606-8501, Japan; 2 Department of High Impact Asia, Grant Management Division, The Global Fund to Fight AIDS, Tuberculosis and Malaria, Chemin de Blandonnet 8, 1214, Vernier Geneva, Switzerland

**Keywords:** HIV, Female drug user, Injecting drug use, Sexual behaviour, Nepal

## Abstract

**Background:**

Women who use drugs are extremely vulnerable to HIV and sexually transmitted infections (STIs), but studies on risk behaviours and HIV infection among female drug users are limited in Nepal.

**Methods:**

In this cross-sectional study conducted between September 2010 and May 2011, HIV prevalence and risk factors for HIV infection were investigated among female drug users recruited in drop-in centres, parks and streets in the Kathmandu Valley. The participants completed face-to-face interviews for a structured questionnaire, HIV pre-test counselling, specimen collection for HIV test and they were provided with their results at post-test counselling.

**Results:**

A total of 269 female drug users were recruited, of whom 28% (n = 77) were found HIV positive; the majority (78%, n = 211) being injecting drug users and aged below 25 years (57%, n = 155). Nearly half (n = 137) of the total participants had shared needles or syringes in the past month, and 131 and 102 participants were involved in commercial or casual sex respectively with only half or less of them having had used condoms in the last 12 months. In multivariate analysis the variables associated with HIV infection included: (a) older age; (b) history of school attendance; (c) frequency of sharing of injection instruments; and (d) unsafe sex with commercial or casual partners.

**Conclusions:**

HIV was highly prevalent among female drug users in the Kathmandu Valley, with its risk being strongly associated not only with unsafe injection practice but also with unsafe sexual behaviours. Awareness raising programmes and preventive measures such as condom distribution, needle or syringe exchange or methadone maintenance therapy should be urgently introduced in this neglected subpopulation.

## Background

The World Drug Report 2013 estimated that in 2011 between 167 to 315 million people aged 15–64 (3.6% - 6.9% of the world’s population in that age group) had used an illicit substance at least once in the previous year [1]. In the same report, the United Nations Office on Drugs and Crime (UNODC) estimated that about 14 million people inject drugs globally and of them 1.6 million (11.4%) are living with HIV. Countries with higher prevalence of HIV infection among people who inject drugs are located in Western and Central Europe, Sub-Saharan Africa, and South and South-East Asia including Nepal [2]. In recent years, there has been a rapid increase in female injecting drug users, especially in Asia and Eastern Europe [3,4]. In Central Asia and the countries like China, India and Russia, drug use and sharing injection equipment is increasing rapidly among females, and in many regions more females are seeking harm reduction services and drug treatment [5,6].

In Nepal, the first case of HIV/AIDS was reported in 1988. As of 2011, national estimates indicated that approximately 50,200 adults and children were infected with HIV, with an estimated overall HIV prevalence of 0.30% in the adult population of 15 to 49 years. Out of the total infections, around one third is estimated to have occurred among females [7]. Although estimated HIV prevalence among Nepal’s adult population is fairly low, the HIV epidemic in Nepal is extremely heterogeneous with respect to the most at risk populations and geographic distribution [8,9]. The epidemic is concentrated in key subpopulations such as commercial sex workers, injecting drug users (IDUs), men who have sex with men, and migrants. IDUs are the subpopulation most heavily affected by the epidemic [10,11]. The drug user population is concentrated in the Kathmandu Valley and in the locations along the East–west Highway, where 30% of all the people living with HIV are IDUs. According to a government report, 42,954 (92.8%) male and 3,356 (7.2%) female drug users were living in the Kathmandu Valley alone in 2007 where 34.8% of IDUs were estimated to be HIV positive [12,13].

Compared to male drug users, information has been limited for female drug users regarding HIV prevalence and their risk behaviours. Though smaller in number, the situation of female drug users could be more serious than that of male drug users for several reasons. Firstly, female drug users are known to trade sex for money or drugs. A study has shown that over half of the female IDUs in China have been involved in sex work [14]. Secondly, female drug users who exchange sex for drugs or cash may not identify themselves at risk of HIV infection because they do not consider themselves as sex workers [15]. Thirdly, condom use can be infrequent among them because drug dependency and financial problems may impair their judgment and power to negotiate for condom use with their sex partners [16-19]. Fourthly, female drug users depend in many cases on male partners for drugs and injections, leading them to an elevated risk of equipment sharing practice [20,21]. Fifthly, female drug users are socially stigmatized more than male drug users, making them hidden and thus it is difficult for them to access preventive services [21-23]. For these reasons, female drug users could expose themselves to exceptional risk of HIV infection and play a critical role in the local HIV epidemic by bridging infection to the broader population through their drug injection network as well as sexual network [18,24,25]. These situations, however, largely remain unknown in Nepal.

With these backgrounds, this study aims to investigate prevalence of HIV infection and social and behavioural correlates of HIV infection among female drug users in Kathmandu Valley, Nepal, with a hope that it may help to improve intervention programmes for female drug users in Nepal.

## Methods

### Setting and sampling procedures

This cross-sectional study was conducted between September 2010 and May 2011 among female drug users of Kathmandu Valley, in which the capital city is situated. Female drug users were recruited in drop-in centres (DICs) that were working for various drug-related harm reduction programmes for female drug users as well as in parks and on the street. Recruitment was carried out directly by ex-drug users and trained outreach workers and indirectly through personal networks. The outreach workers first mapped out the groups and areas where they expect to encounter the target population, and they then set out to actively recruit potential candidates there. The time of field observations varied to cover morning, afternoon, evening, and late night hours. They often made use of existing social networks within a population. As selection proceeds, suitable new candidates with appropriate characteristics or behaviour were sought within the social networks of respondents already included in the study (snowball sampling) [26]. The inclusion criteria for the study was that they were current drug users of at least 16 years old and willing to give informed consent for both questionnaire survey and HIV testing. Eligible respondents received an explanation about the survey, its purpose and types of the questions to be asked.

### Interview

Trained outreach workers and peer educators of the HIV/AIDS programme who had already established trust with female drug users through their daily activities interviewed participants face to face using structured questionnaires. A small incentive was provided to each participant for travel and refreshment. The name of the participants or their addresses were not recorded anywhere. Instead, they were provided with a unique identification (ID) number written on a plastic-coated card that was used for both questionnaire and HIV testing. Same ID was used for pre- and post-counselling and for the provision of test results.

### Instrument

The structured questionnaire was developed from a questionnaire recommended and produced by Family Health International for IDUs [27]. With modifications made following the findings of the preliminary qualitative study (conducted among 21 female drug users in May 2010), the final questionnaire included 90 questions. The topics in the questionnaire included the main socio-demographic characteristics, drug use practices, frequency and duration of injection drug use, sexual activities and a history of sexually transmitted infections (STIs), and knowledge and perception of HIV/AIDS. Questions on knowledge included: (1) Can people protect themselves from HIV by using condoms?; (2) Can a person get HIV from mosquito bites?; (3) Can people protect themselves from HIV by having one uninfected faithful sex partner?; (4) Can people protect themselves from HIV by abstaining from sexual intercourse?; (5) Can a person get HIV by sharing a meal with someone who is infected?; (6) Can a person get HIV through injections with a needle that was already used by someone else?; (7) Can people who inject drugs protect themselves from HIV by switching to non-injecting drugs?; (8) Can a pregnant woman infected with HIV transmit the virus to her unborn child?; and (9) Can a woman with HIV transmit the virus to her newborn child through breastfeeding? All of these questions were given in a Likert scale with options of “yes”, “no” and “do not know”.

### HIV testing

After completing the interview, blood samples were collected from each study participant by finger prick blood sampler complying with the National HIV Testing Protocol. Considering the availability of limited human and financial resources, the Ministry of Health and Population of Nepal has recommended the use of two or more rapid tests based on different test principles (antigens) as a minimum standard HIV test algorithm to be followed at all levels of the health care delivery system [28]. Two rapid test kits Determine HIV 1/2 and Uni-Gold HIV 1/2 were used for the purpose. The initial screening test was performed using Determine HIV 1/2 and then retested using the Uni-Gold HIV 1/2 when the first test result was positive for confirmation. A third test SD Bioline HIV 1/2 was performed for final confirmation when there was discrepancy in the first two tests. If the third test showed a reactive result, the tested sample was reported as “HIV positive” and if the third test showed a non-reactive result, it was reported as “HIV negative”. Sensitivity and specificity of combined testing algorism of Determine and SD Bioline are reported to be both 100% [29,30]. The HIV test results were kept confidential. Pre-test counselling was provided as a part of informed consent and the participants were informed of the testing result only at post-test counselling.

### Ethical issues

This research protocol was approved by the Nepal Health Research Council and the Committee for Research on Human Subjects at Kyoto University in Japan. Separate written informed consents were obtained for the interview and HIV testing, and no personal identifier was recorded on the questionnaires. All the participants were advised to undertake free HIV testing for final clinical diagnosis.

### Statistical analysis

Statistical analyses were carried out using SPSS software for Windows (version 19, IBM Inc., Chicago). Bivariate analyses were conducted to estimate the association of demographic or behavioural variables with HIV infection, calculating crude odds ratio (OR) with 95% confidence interval (CI). Multiple multivariate models were run: (a) to compare the predictive powers of behavioural variables of different time frames; and (b) to show independent correlates to HIV infection. The significant variables from bivariate analyses, epidemiologically important variables and synthetic variables created by combining related variables were included in the models. Independent variables were assessed for multicollinearity, variance inflation factor and tolerance statistics were within acceptable limits for all variables [31]. From multivariate models adjusted odds ratio (AOR) and 95% CI were calculated. A *p*-value less than 0.05 (2-sided) was considered to be statistically significant. Answers to the HIV knowledge questions were transformed into scores by giving 1 for correct answer and 0 for otherwise.

## Results

A total of 269 participants were included in the analysis, of whom 28.6% were HIV positive. The major socio-demographic characteristics of the participants are shown in Table 1.

**Table 1:** Socio-demographic characteristics of female drug users with or without HIV infection recruited in the Kathmandu Valley, Nepal

	**Positive HIV test (n = 77) n (%)**	**Total (n = 269)**	**Crude OR (95% CI)**	***p *** **value**
**Place of recruitment**				
Street/Park	40 (28.6)	140	1.00	
Drop in centre	37 (28.7)	129	1.01 (0.59-1.71)	0.984
**Age (years)**				
16 - 19	7 (25.9)	27	1.00	
20 - 24	25 (19.5)	128	0.69 (0.26-1.82)	0.457
25 - 29	28 (35.0)	80	1.54 (0.58-4.08)	0.387
30 - 38	17 (50.0)	34	2.86 (0.96-8.52)	0.060
Mean (SD) (Median)	26.3 (6.0) (25.0)	24.4 (4.8) (23.0)	1.12 (1.06-1.18)	<0.001
**Marriage and live-in partnerships**				
Not married, not living with sexual partner	6 (9.4)	64	1.00	
Not married, living with sexual partner	25 (32.5)	77	4.65 (1.77-12.22)	0.002
Currently married, not living with spouse or any other sexual partner	12 (36.4)	33	5.52 (1.84-16.59)	0.002
Currently married, living with spouse or other sexual partner	34 (35.8)	95	5.39 (2.11-13.79)	<0.001
**Level of education**				
Never went to school	2 (4.1)	49	1.00	
Primary (1–5 years)	15 (48.4)	31	22.03 (4.54-107.04)	<0.001
Secondary (6–10 years)	26 (27.4)	95	8.86 (2.01-39.10)	0.004
Higher (11 years and higher)	34 (36.2)	94	13.32 (3.04-58.28)	0.001
**Job situation**				
Have a job	32 (25.6)	125	1.00	
Jobless	45 (31.3)	144	1.32 (0.77-2.25)	0.307

SD, standard deviation.

OR, odds ratio.

CI, confidence interval.

More than half (52.0%) of the study participants were recruited in the streets. No association was detected between sites of recruitment and HIV status of the participants (*p* = 0.98). More than half of the participants (57.6%) were under 25 years old; the median age being 23 years. HIV infection was found to be associated with older age groups; the prevalence being 50.0% in the 30 years and above. The majority (76.2%) of the participants were married and/or cohabitating with their sexual partners, which is highly associated with HIV infection (*p* = 0.002 or *p* < 0.001). More than 80% of the participants had ever been to school, and the HIV prevalence (34.1%) is much higher in this group than those without school education (4.1%) (*p* = 0.001). Although 53.3% of the participants were unemployed, the job situation of the participants was unrelated to the HIV status (*p* = 0.307).

Table 2 shows the prevalence of risky behaviours in this population and the results of bivariate analyses between HIV status and HIV/STI knowledge score or behavioural variables. Risky behaviours are highly prevalent in this population. Injection practice in lifetime was reported from 78.4% (211/269); of them 24.2% (51/211) shared needles and/or syringes most times or always, and 86.7% (183/211) had shared a cooker or other utensils sometimes or more with other people in the past one month. Sex with regular, non-regular non-commercial partners (herein after “casual partners”) and commercial partners were reported by 74.1% (166/224), 45.5% (102/224) and 58.5% (131/224) of sexually active participants respectively. Among them 79.5% (132/166), 75.5% (77/102) and 87.8% (115/131) reported that they are not always using condoms, respectively.

**Table 2:** Bivariate association of behavioural factors with HIV infection among female drug users recruited in the Kathmandu Valley, Nepal

	**Positive HIV test (n = 77) n (%)**	**Total (n = 269)**	**Crude OR (95% CI)**	***p *** **value**
**HIV Knowledge scores**				
6 - 9	66 (27.8)	237	1.00	
0 - 5	11 (34.4)	32	1.36 (0.62-2.97)	0.445
**Drug use behaviour**				
Ever injected illegal/non-medical drugs				
No	8 (13.8)	58	1.00	
Yes	69 (32.7)	211	3.04 (1.37-6.76)	0.006
Ever used a needle or syringe previously used by someone else				
No/Non-IDU	23 (17.4)	132	1.00	
Yes	54 (39.4)	137	3.08 (1.75-5.43)	<0.001
Frequency of injecting with a needle or syringe previously used by someone else in the past one month				
Non-IDU/Never	24 (17.5)	137	1.00	
Occasionally/About half the time	36 (44.4)	81	3.77 (2.02-7.01)	<0.001
Always/Most times	17 (33.3)	51	2.35 (1.13-4.89)	0.022
Frequency of sharing cooker/vial/container, cotton/filter, or rinse water when injecting in the past one month				
Never/Non-IDU	19 (22.1)	86	1.00	
Sometimes	17 (21.8)	78	0.98 (0.47-2.06)	0.963
Always/Often	41 (39.0)	105	2.26 (1.19-4.30)	0.013
Frequency of injecting drugs in the past one month				
Non-IDU	8 (13.8)	58	1.00	
6 times a week or LESS	9 (15.5)	58	1.15 (0.41-3.22)	0.793
1 time a day or MORE	60 (39.2)	153	4.03 (1.79-9.10)	0.001
**Sexual behaviour**				
Ever had sexual intercourse				
No	2 (4.4)	45	1.00	
Yes	75 (33.5)	224	10.82 (2.55-45.89)	0.001
Sexual intercourse in the last 12 months with any type of partner				
No/Never had sex	4 (5.9)	68	1.00	
Yes	73 (36.3)	201	9.13 (3.19-26.08)	<0.001
Sexual intercourse with regular partner in the last 12 months				
No/Never had sex	15 (14.6)	103	1.00	
Yes	62 (37.3)	166	3.50 (1.86-6.58)	<0.001
Sexual intercourse with commercial partner in the last 12 months				
No/Never had sex	19 (13.8)	138	1.00	
Yes	58 (44.3)	131	4.98 (2.75-9.02)	<0.001
Sexual intercourse with non-regular non-commercial partner in the last 12 months				
No/Never had sex	25 (15.0)	167	1.00	
Yes	52 (51.0)	102	5.91 (3.32-10.51)	<0.001
Frequency of condom use in the last 12 months with all regular partners				
Never had sex/No sex with regular partner in the last 12 months	15 (14.6)	103	1.00	
Always/Often	20 (32.3)	62	2.79 (1.30-6.00)	0.008
Sometimes/Never	42 (40.4)	104	3.97 (2.03-7.79)	<0.001
Frequency of condom use in the last 12 months with all commercial partners				
Never had sex/No sex with commercial partner in the last 12 months	19 (13.8)	138	1.00	
Always/Often	36 (54.5)	66	7.52 (3.79-14.91)	<0.001
Sometimes/Never	22 (33.8)	65	3.20 (1.58-6.49)	0.001
Frequency of condom use in the last 12 months with all non-regular non-commercial partners				
Never had sex/No sex with casual partner in the last 12 months	25 (15.0)	167	1.00	
Always/Often	20 (45.5)	44	4.73 (2.28-9.82)	<0.001
Sometimes/Never	32 (55.2)	58	6.99 (3.58-13.66)	<0.001

Non-IDU, non-injecting drug user.

OR, odds ratio.

CI, confidence interval.

All these variables are significantly associated with increased risk of HIV infection except for HIV knowledge. The prevalence of HIV is high among those who had ever injected (OR = 3.04, *p* = 0.006) as well as among those who ever used needle or syringes previously used by someone else (OR = 3.08, *p* < 0.001). Frequency of injection in the past one month is associated with positive HIV status in a dose-dependent manner; the participants injecting once or more a day have the highest (39.2%) HIV prevalence (OR = 4.03, *p* = 0.001). Frequency of sharing injection instruments or other accessory utensils such as a cooker in the past one month is also associated with positive HIV status but not in a dose-dependent manner. Sexual experiences, both over a life time and in the last 12 months, are strongly associated with HIV status with equivalent magnitudes (OR = 10.82 and 9.13, *p* ≤ 0.001). Similarly, sexual experience with any type of partner in the past 12 months is strongly associated with the HIV status (OR = 3.50-5.91, *p* < 0.001) with the highest association being with casual partners. The majority of the participants used condoms inconsistently with any type of sexual partner. The frequency of condom use with a regular partner or casual partner is associated with HIV status in a dose-dependent manner. The prevalence is highest among the participants who never or only sometimes used condoms with their regular partners (40.4%, OR = 3.97, *p* < 0.001) or casual partner (55.2%, OR = 6.99, *p* < 0.001). The frequency of condom use with commercial partners is also significantly associated with the HIV status but not in a dose-dependent manner.

Table 3 summarizes the results of multivariate analyses of factors associated with HIV infection. Multiple logistic regression analyses were carried out using behavioural variables with different time frames as well as synthetic variables with different risk specificities. This analytic strategy was adopted because although some questions were asked in an “ever” time frame for injection and sexual behaviours, the rest of the questions were asked in different time frames; “in the past one month” for drug use and “in the past 12 months” for sexual behaviours. It seemed therefore necessary to assess the effect of the time frame on the predictive power of the variables. It was also expected that the predictive power of the variables would be enhanced by creating synthetic variables to represent more directly the risk of behaviours.

**Table 3:** Multivariate association of behavioural factors with HIV infection among female drug users in the Kathmandu Valley, Nepal

	**All drug users**						**Only IDUs**
	**Model 1**	**Model 2**	**Model 3**	**Model 4**	**Model 5**	**Model 6**	**Model 7**
	**Adjusted odds ratio**						
	**(95% confidence interval)**						
Age	1.10*	1.10*	1.11*	1.11*	1.07*	1.07	1.08*
	(1.04-1.17)	(1.03-1.17)	(1.04-1.18)	(1.04-1.19)	(1.01-1.15)	(1.00-1.15)	(1.00-1.17)
Ever attended school (ref. No)	10.85*	9.03*	10.83*	10.52*	6.14*	6.21*	4.85
	(2.46-47.79)	(2.05-39.73)	(2.44-48.13)	(2.38-46.56)	(1.34-28.06)	(1.31-29.49)	(0.96-24.55)
Currently married, or currently not married but living with sex partner (ref. Not married, not living with sexual partner)	2.33	2.15	1.84	1.85	2.01	1.62	1.69
	(0.72-7.53)	(0.78-5.94)	(0.57-5.96)	(0.57-6.01)	(0.72-5.66)	(0.57-4.62)	(0.52-5.47)
Ever injected illegal/non-medical drugs (ref. No)	2.48*	2.41*			2.65*		
	(1.05-5.84)	(1.02-5.73)			(1.08-6.49)		
Ever used a needle or syringe previously used by someone else (ref. No/Non-IDU)			2.50*				
			(1.35-4.63)				
Used needles or syringes previously used by someone else in the past one month (ref. Never/non-IDU)				2.60*			
				(1.40-4.81)			
Used needles or syringes previously used by someone else, injecting 6 times a week or less in the past month (ref. Non-IDU/Never)						0.93	0.78
						(0.24-3.64)	(0.19-3.26)
Used needles or syringes previously used by someone else, injecting 1 time a day or more in the past month (ref. Non-IDU/Never)						2.53*	2.19
						(1.28-4.99)	(0.99-4.84)
Ever had sexual intercourse (ref. No)	2.52		2.72	2.57			
	(0.40-15.96)		(0.43-17.20)	(0.41-16.26)			
Had sexual intercourse in the last 12 months (ref. No/Never had sex)		3.77*					
		(1.19-11.97)					
Had sexual intercourse with regular partner in the last 12 months (ref. No/Never had sex)					1.24		
					(0.57-2.70)		
Had sexual intercourse with commercial partner in the last 12 months (ref. No/Never had sex)					2.20*		
					(1.09-4.48)		
Had sexual intercourse with non-regular non-commercial partner in the last 12 months (ref. No/Never had sex)					2.49*		
					(1.25-4.95)		
Frequency of condom use in the last 12 months with all regular partner(s)^a^							
Always/Often						1.15	1.13
						(0.45-2.93)	(0.40-3.20)
Sometimes/Never						1.33	1.19
						(0.57-3.11)	(0.47-3.04)
Frequency of condom use in the last 12 months with all commercial partner(s)^a^							
Always/Often						3.35*	3.41*
						(1.47-7.61)	(1.39-8.40)
Sometimes/Never						1.45	1.66
						(0.63-3.35)	(0.67-4.08)
Frequency of condom use in the last 12 months with all non-regular non-commercial partner(s)^a^							
Always/Often						1.71	2.13
						(0.72-4.07)	(0.82-5.53)
Sometimes/Never						3.11*	3.11*
						(1.36-7.13)	(1.28-7.56)

IDUs, injecting drug users.

^a^ref. Never had sex/No sex with this type of sexual partner in the last 12 months.

*p value <0.05.

Models 1 to 6 include all drug users but model 7 includes only injecting drug users. “Ever injected illegal/non-medical drugs” and “Ever had sexual intercourse” were entered in model 1, while the latter was replaced with “Had sexual intercourse in the last 12 months” in model 2 showing that association of sexual behaviour is more prominent “in the last 12 months” than “ever”. In models 3 and 4, on the other hand, a variable “Ever injected illegal/non-medical drugs” was replaced with a variable “Ever used needles or syringes previously used by someone else” (model 3) or a synthetic variable “Used needles or syringes previously used by someone else in the past one month” (model 4), while keeping the variable “Ever had sexual intercourse” the same as in model 1. From the comparison of models 1 to 4, it was shown that all injection variables had equivalent predictive power (AOR = 2.5) irrespective of time frame or the presence/absence of sharing practice, and that sexual variables had predictive powers that were equivalent to or more potent than the injection variables among the participants.

Model 5 is an extension of model 2 where the risks of sexual intercourses with different types of partners in the past 12 months with HIV infection were compared. While significant association was detected for sexual intercourses with commercial and casual partners, it was not significant with regular partners. Model 6 is the extension of model 4 using a synthetic variable where “Used needles or syringes previously used by someone else in the past one month” was combined with the frequency of injection in the past month. It was shown that frequent unsafe injection practice (sharing needles and syringes once a day or more) and frequent unsafe sex (intercourses with ‘sometimes or never’ condom use) with casual partners were significantly associated with HIV infection, while with commercial sexual partners intercourses with ‘always or often’ rather than infrequent condom use showed significant association. Such an association pattern was maintained when analysis was confined to female injecting drug users (model 7). Throughout these models, age and “Ever attended school” but not “Marital status” were significantly associated with HIV infection. “Ever attended school” was the most potent predictor of HIV infection, while its association nearly halved when sexual behaviour variables were switched from the general ones to those specific to sexual partners. Behavioural and injecting practice variables were introduced in the order of all possible combinations into models 5 through 7, yielding similar results.

## Discussion

Overall, HIV prevalence in this population was revealed to be 28.6%; 33% in those who practised injecting drug use and 14% even among those who reportedly never experienced injecting drug use. In Nepal, the Integrated Biological and Behavioral Surveillance (IBBS) surveys are conducted at regular intervals among male IDUs. The latest round of IBBS showed a significant decline in HIV prevalence among male IDUs in Kathmandu Valley, from 51.7% in 2005 to 6.3% in 2009 [10], which is much lower than the HIV prevalence we found among female IDUs. This study strongly suggests that female drug users are now one of the most vulnerable populations with regard to HIV infection in Nepal.

Consistent with previous studies, injection behaviour showed a strong association with HIV infection in this population. This association could be causal because the sharing of injection instruments was associated strongly with HIV infection and in a dose-dependent manner. A study in Montreal among active drug users found that increasing injection frequency is highly correlated with HIV transmission, as it may reduce the chances of sterile injecting equipment being used each time [32]. Also, where group injecting is common, women may be the last to use the needles/syringes [33]; this may have strong implications with regard to the spread of HIV.

Unprotected sexual behaviour was also of great risk in this study. Risk significantly increased for sex with commercial or casual sex partners. The association of HIV infection and sex with commercial partners was not dose-dependent, where the people who reported more condom use were more likely to be HIV positive. The reason for this association could be multiple. It may be that participants who were involved in commercial sex and knew their HIV infection before the study might have provided socially desirable answers to our interview, or they were really using condoms to either prevent HIV transmission to their clients or as self-protection from re-infection. A recent study by UNODC among female drug users in Nepal has reported nearly 90% of condom use in last sex act with commercial sex partners [34]. In addition, previous studies among drug users have found safer sex practices strongly associated with HIV infection. A study in the United States found that self-reporting of being HIV-infected was the strongest factor associated with consistent condom use in the past 6 months [35]. Another study in Puerto Rico found that HIV-positive drug users were nearly five times more likely to use condoms during vaginal sex [36]. In contrast, HIV infection risk of the sex with casual partners was dose-dependent on condom use, where the people who have less condom use are more likely to be HIV positive. Studies in other countries have observed the association between sex with casual partners and HIV infection among IDUs [37]. These women may not have skills to negotiate for condom use, and even if the woman is aware of her HIV status, in casual relationships she may not feel responsible for preventing further transmission of HIV to this type of partner.

Injection or sexual behaviours at high risk of HIV infection were shared by around 70% or more participants with HIV infection and even one third to half of participants without HIV infection in this study. This strongly suggests that if left uncontrolled the HIV epidemic could expand in this population and that this population could continue to be a source of HIV infection through its networks of injection and/or behavioural practices.

It is increasingly being recognized that women who use drugs are generally different from men who use drugs, and thus have different needs [6,38-40]. Targeted HIV prevention and treatment programmes should be urgently developed and implemented for this population. Provision of clean needles and syringes or methadone maintenance therapy (MMT) will be useful for the prevention of HIV transmission through injecting network. Several studies have shown that drug users who used needle and syringe exchange programs were less likely to share needles and syringes [41,42]. Programs such as needle and syringe exchange and MMT can achieve high coverage of IDUs in some settings [42]. A study in Amsterdam showed the benefits of the combined availability of needle exchange and MMT, and argued that involvement with both services, compared to only one, was associated with a lower incidence of HIV infections among IDUs [43]. Additionally, free STI diagnosis and treatment and condom distribution will be suitable for the prevention of HIV infection through sexual network [44]. Despite mixed evidence from three large community-based randomized controlled trials in sub-Saharan Africa, syndromic and mass treatment of STIs may contribute to HIV infection prevention [44]. Finally, problems of drug use and commercial/casual sex are frequently intertwined in female drug users as they often engage in commercial/casual sex to get drugs or money [45,46]. An overlap of sex and drug networks among IDUs enhances their vulnerability to HIV infection and promotes HIV transmission among their sexual partners. MMT, the most widely available treatment for opiate addiction, is of particular importance since it can prevent infection through both injecting drug use and commercial/casual sex for drugs at the same time. MMT has shown to reduce both injection drug use and the risk of infection with HIV [47,48]. Since the size of this subpopulation has been unknown, these measures should be accompanied with studies to estimate the size of this population.

In this study, most of the behavioural questions were taken from the standard questions of Family Health International [27]. In this set of questions drug use and sexual behaviours are asked, though in part covering the participant’s lifetime, but mostly in different time frames; one month and one year for drug use and sexual behaviours, respectively. Since such a difference in time frames could influence the predictive power of the variables, we compared their predictive powers and found that predictive powers were largely independent of time frame for both types of behaviours, suggesting that once initiated, the same behavioural pattern will be maintained over a long period of time in this population.

Older age and having education remained as strong predictors of HIV infection even in the presence of all behavioural variables that could affect HIV infection. Since HIV infection other than through injecting drug use and sexual intercourse is unlikely in this population, this may suggest the uncertainty of behavioural variables or that the behavioural variables we used could reflect the risk for HIV infection only partially. The uncertainty of information could come from self-reported nature of the information which could be affected by recall bias, the psychotic effect of drugs or by socially desirable answers to the sensitive and/or illegal behaviours.

One of the limitations of this study is the sampling procedure because it was not random; the participants may not be a true representative to the female drug user population in the study region. Participants were recruited through a snowballing process. Though efforts were made to recruit the initial respondents in many locations and opportunities to ensure variability, our sampling procedure could still introduce the bias into the participants in a way that people who have larger social connections and have similar characteristics to initial respondents are overrepresented. To get an unbiased prevalence of HIV infection and risky behaviours, more sophisticated sampling methods, such as respondent driven sampling [49-51] should be considered. Finally, cross-sectional design of this study limits the causal inference in association detected in this study.

## Conclusions

HIV was highly prevalent among female drug users in Kathmandu Valley, with its risk being strongly associated not only with unsafe injection practice but also with unsafe sexual behaviours. Awareness raising programmes and preventive measures such as condom distribution, needle or syringe exchange or methadone maintenance therapy should be urgently introduced in this neglected subpopulation.

## Abbreviations

IDUs: Injecting drug users; DICs: Drop-in centres; ID: Identification; STIs: Sexually transmitted infections; OR: Odds ratio; CI: Confidence interval; AOR: Adjusted odds ratio; IBSS: Integrated bio-behavioural surveillance survey; NCASC: National Center for AIDS and STD Control; MMT: Methadone maintenance therapy.

## Competing interests

The authors declare that they have no competing interests.

## Authors’ contributions

BG, SZ, MOK, and MK participated in the conception and design of the study. BG supervised the data collection. BG, SPS and MK performed the statistical analysis and interpretation of results. BG and MK drafted the manuscript. All authors revised, read and approved the final manuscript.

## Pre-publication history

The pre-publication history for this paper can be accessed here:

http://www.biomedcentral.com/1471-2458/13/1238/prepub
